# 

**Published:** 1892-10-08

**Authors:** 


**Notices of Births, Deaths, and Marriages,** are inserted in this column at a charge of 2s. 6d. for an announcement not exceeding four lines.

### NOTICE TO CORRESPONDENTS.

All MS., Letters, Books for Review, and other matters intended for the Editor should be addressed The Editor, The Lodge, Porchester Square, London, W.

The Editor cannot undertake to return rejected M.S., even when accompanied by stamped directed envelope.

**Notice to Advertisers and Subscribers.**

All Advertisements, Orders for Copies of The Hospital, and Business Communications should be addressed to The Publisher, *not to the Editor*, The Hospital, 140, Strand, W.C.

The Cost of Subscription, post free, is as follows:—Per week, 2½d.; per quarter, 2s. 6d.; per half-year, 5s.; per annum, 8s. 6d. Foreign:—Per annum, 10s. 8d.; payable, in all cases, in advance.

**"Burdett's Hospital Annual," 1891—1892.**

Third year of publication. 600 pp. Boards, gilt lettering. A most complete guide to British and Colonial Hospitals, Dispensaries, Nursing and Convalescent Institutions, and Asylums. Price 3s. 6d. Published by The Scientific Press (Limited), 140, Strand, W.C.

**NOTICE.—The Index for Vol. XI. (ending March, 1892) is on sale at the Office of this Journal, price 2½d., post free.**

### THE VICTORIA HOSPITAL FOR CHILDREN.

### SCRAPS AND GLEANINGS.

Lord Winmarleigh has bequeathed £500 to the Warrington Infirmary.

The sum of £19 2s. 5½d. was collected at Wantage on Hospital Sunday for the Cottage Hospital.

Mr. Simmonds of Southport, promises to give £20,000 to the county of Cumberland for hospital purposes.

The foundation-stone of the new Southport Infirmary is to be laid by the Mayor of Southport on Saturday, October 29th.

A little girl of four years old has been crushed to death by a heavy glass case falling upon her when she was at play in the street.

A sum of £[illegible]41 8s. 8d. has been received by the Committee of the Victoria Hospital for Children as a result of the recent trades and friendly societies' demonstration.

Ripe strawberries were gathered last week from gardens at Bekesbourne, near Canterbury, and also in the neighbourhood of Ashford. The fruit was luscious and full-grown.

Sir John Arnott has offered a hulk to the Cork Rural Health Committee, and to put it in a sufficient state of repair so that it may be used for the purpose of a floating hospital.

The Duke of Connaught, while out shooting at Balmoral a week ago, sprained his knee. His Royal Highness was in consequence unable to fulfil his engagement to open a new infirmary at Aberdeen on Tuesday last.

The largest cheese ever made is to be exhibited among the Canadian dairy products at the World's Fair, Chicago. The cheese weighs over 22,000 pounds. It contains the curd of one day's milk supplied by 10,000 cows.

The outbreak of typhoid fever at the village of Easington has been traced to the impure water supply. The local sanitary authority have taken the matter in hand, and ordered that the wells shall all be thoroughly cleaned out within fourteen days.

The number of new patients admitted during the year to the Ramsgate and St. Lawrence Dispensary was 1,937; at the last annual report the number of patients on the books was 21[illegible]; of these 1,8[illegible]0 were cured, 34 relieved, 51 died, and [illegible]55 remain on the books.

At a meeting of the delegates of the Friendly and Trades Societies' Demonstration Committee, Watford, it was decided to divide the money collected as follows: £70 to the Cottage Hospital, £20 to the Herts Convalescent Home, and £20 to the Watford Nurse Fund.

At the monthly meeting of the directors of the Longton Cottage Hospital, a portion of the Library Fund was ordered to be spent in the purchase of more books and the best thanks of the meeting was accorded to the Mayor of Longton for his valuable gift of books and book-cases.

At the monthly meeting of the West Kent Joint Hospital Board it was agreed to open, if necessary, the isolation wards for the reception of scarlet fever patients. At present they are used only for diphtheria cases, and there is no accommodation for small-pox patients should any arise.

The Duke of Edinburgh, in presenting the certificates to the successful students of the Great Western Railway Branch of the Exeter Centre of the St. John's Ambulance Association, remarked how necessary it was that all railway servants should be able to render first aid to the injured.

Mr P. D. Longstaff, M.D., father of Dr. Longstaff, member of the London County Council, has died at Wandsworth at the age of ninety-four. He built and presented to Wandsworth the Longstaff wing of the Free Library. Quite recently, mainly owing to Dr. Longstaff's munificence, that institution was freed from a debt of four thousand pounds.

The proposed naval gift to Princess Marie of Edinburgh on her marriage is to be a silver inkstand. The base is ebony, on which stands the silver inkstand on four lions; the ink-pots surmounted by naval crowns, and in the centre is the figure of Britannia on the shield, with the English, Russian, and Roumanian flags in coloured enamel. The whole will cost about £8[illegible].

The Duke of Edinburgh recently inspected the Royal Naval Hospital, Stonehouse, he was accompanied by Flag-Lieutenant Colin Keppel and Mr. Henry Rickard. His Royal Highness was met by Inspector-General Lloyd and the officers of the staff, who escorted him through the various wards, the Duke taking much interest in the nursing and condition of the patients.

Dr Jaromer, Baron of Mundy, the founder and chief physician of the Vienna Retungs Gesellschaft, has just attained his seventieth year. Baron Mundy has been the means of introducing many of the modern improvements for the transport and comfort of the sick and wounded on the battle field. He was formerly an army officer, and has devoted his life and service to the ambulance service.

A serious difficulty has arisen on the Tyne as to the burial of persons who may die of cholera in the Floating Hospital. It is impossible that they should be buried at sea, because the trawlers fish as far as seventy miles out, and the bodies would be brought up with the fish. The hospital lies off Yarrow, about six miles from Newcastle, but the inhabitants of both towns object to the bodies being carried through their streets to the cemeteries.

An iron hospital is being shown at the Portsmouth Health Exhibition. One of the wards has been furnished by the Royal Portsmouth Hospital Staff in order to show the advantage such a building possesses in dealing with epidemics. A few convalescent child patients have been taken there, and nurses are in daily attendance from the hospital. Should an accident occur in the exhibition it would at once be treated in this ward, where all necessary appliances are in readiness.

Patients will be received into the new Fever Hospital, Stamford Hill, on the 8th inst. There are about fifty large wood buildings, standing 100 feet apart and connected by covered ways 2,120 feet in length; with raised platforms forming a quadrangle. The new hospital has accommodation for 456 patients, allowing 144 superficial feet of area to each bed, but if the area is limited to 120 superficial feet there will be sufficient room for 548 patients. The resident staff amounts to 207 persons. The total accommodation therefore is 755.

### THE STUDENT OF MEDICINE.

[All communications for this Supplement should be addressed to The Editor of The Hospital, at 140, Strand, with the words, "Students' Column," written in the left hand top corner of the envelope.]

### APPOINTMENTS.

Bluett, J., M.R.C.S., L.M., appointed Medical Officer of Health for the Borough of Chesterfield. Bostock, J., M.R.C.S.Eng., L.S.A., appointed Medical Officer for the Wymeswold District of the Loughborough Union. Bratton, J. A., M.A.Cantab, L.R.C.P.Lond., M.R.C.S.Eng., appointed Medical Officer of the Ewell District of the Epsom Union. Browne, Dr., appointed Second Assistant Surgeon to the Derby Friendly Societies' Medical Association. Burns, R. J., L.R.C.P.Lond., M.R.C.S.Eng., appointed, pro tem., Surgeon to the Sunderland Police. Byass, E. S., M.B., C.M., L.R.C.S.Edin., re-appointed Medical Officer for the No. 2 District of the Cuckfield Union. Cowan, R. H., M.R.C.S., L.S.A., appointed Honorary Medical Officer to Royal Albert Edward Infirmary, Wigan. Dalison, B. E., appointed Medical Officer and Public Vaccinator for the Puddletown District of the Dorchester Union. Drew, C. L., M.B., M.R.C.S.Eng., appointed Coroner for West Middlesex. Holman, H. C., M.R.C.S., re-appointed Medical Officer for the No. 2 Sanitary Districts of the Hailsham Union. Hoskins, E., M.R.C.S., re-appointed Medical Officer for the Little Eaton Sanitary District of the Shardlow Union. Hyatt, J. T., L.R.C.P.Edin., M.R.C.S., appointed District Medical Officer of the Shepton Mallet Union. Innes, J. C., L.R.C.S., L.M.Edin., appointed Medical Officer for the Cromford District of the Bakewell Union. Jackson, A. M., M.D.Oxon., M.R.C.S.Eng., appointed Senior Assistant Medical Officer to the Kent County Asylum, near Maidstone. Jackson, R., M.B., C.M.Edin., appointed Deputy Medical Officer of the Sutton District of the Prescott Union. Knox, J., M.D.Aberd., L.R.C.S.Edin., appointed Medical Officer for the Hartington Middle Quarter District of the Bakewell Union. Moon, D. S., L.R.C.P., L.R.C.S.Edin., appointed Honorary Consulting Surgeon to the Royal Infirmary, Dundee. Moore, S. J., M.D.Glas., appointed Consulting Medical Officer to the Caledonian Railway Company. Pritchard, E. J., M.B., C.M.Glasg., re-appointed Medical Officer for the Stoke Golding District of the Hinckley Union. Purvis, G. C., M.D., B.Sc. (Pub. Health) Edin., appointed Assistant Medical Officer of Health (temporary) to the Port of Leith. Readman, T., L.R.C.P.E., L.R.C.S.E., and L.F.P., and S.G., and L.M.E., appointed Medical Officer and Public Vaccinator for the Aldbrough District of the Skirlaugh Union. Shortridge, T. W., M.D.Brux., L.R.C.P., L.R.C.S.Edin., re-appointed Medical Officer of Health to the Honiton Town Council. Smith, R. B., L.R.C.P.Edin., L.R.C.S.Irel., re-appointed Medical Officer for the Wolvery District of the Hinckley Union. Stockwell, F., M.D.Lond., M.R.C.S.Eng., appointed District Medical Officer to the Shepton Mallet Union. Stoney, R., appointed Surgeon to the Peninsular and Oriental Steam Navigation Company's service. Strover, H. C., L.S.A., L.A.H.Dub., appointed Medical Officer of the Sandy District of the Biggleswade Union. Thompson, Dr. S., appointed Medical Officer for the Buckland District of the Bideford Union. Welsh, R. C., M.B., C.M.Edin., appointed Medical Officer of the Tempsford District of the Biggleswade Union. Williams, F. M., L.R.C.P., L.M.Edin., M.R.C.S.Eng., D.P.H.Camb., appointed Medical Superintendent of Quarantine at Port of Plymouth. Williamson, A. S., M.A., M.B., C.M.Aberd., appointed Medical Officer of Health to the Sandal Local Board. Wilmott, C. C. E. M.B.Durh., appointed Junior Assistant Medical Officer of Health to the Middlesex County Asylum, near Tooting, S.W.

### VACANCIES.

The following vacancies are announced this week, the amount of salary in each case being given after the name of the institution:

### Resident Medical Officers.

Bethlem Hospital, S.E., two Resident Clinical Assistants—Apartments rations, and attendance provided.

Clayton Hospital and Wakefield Dispensary, Wakefield, Junior House Surgeon; unmarried—Honorarium £40 per annum, with board, lodging, and washing.

### THE STUDENT OF MEDICINE—(*continued*).

Coventry and Warwickshire Hospital, 10, Hay Lane, Coventry, Assistant to the House Surgeon.—Appointment for six months. Honorarium, £15 with board, &c.

General Hospital, Birmingham, House Physician—Salary £70 per annum.

Holborn Union, Resident Medical Officer for the Workhouse, Shepherdess Walk, City Road, N.—Salary £200 per annum, with furnished apartments, gas, coal, and washing.

Hospital for Consumption and Diseases of the Chest, House Physician.

Kent and Canterbury Hospital, Assistant House Surgeon; unmarried—Salary £50 per annum, with board and lodging.

Kent County Lunatic Asylum, Barming Heath, near Maidstone, Third Assistant Medical Officer; unmarried—Salary £175 per annum, rising £5 yearly to £200.

Kimberley Hospital, Kimberley, South Africa, Senior House Surgeon.—Salary £750, with quarters.

Royal United Hospital, Bath, House Surgeon; must be M.R.C.S.Eng., appointment for one year.—Salary £60 per annum, with board, &c.

### Non-Resident Medical Officers.

Brighton Throat, and Ear Hospital, 23, Queen's Road, Brighton, Non-resident House Surgeon.—Salary at the rate of £50 per annum.

Charing Cross Medical School, Lecturer on Organic Chemistry.—Remuneration guaranteed minimum of £100 per annum.

City of London Hospital for Diseases of the Chest, Victoria Park, E., Pathologist—Salary 100 guineas per annum.

County Borough of Stockport, Medical Officer of Health for the District of the Borough, and to the Infectious Diseases Hospital, and Surgeon to the Police—Salary £400 per annum.

General Hospital, Birmingham, Pathologist—Salary £120 per annum.

General Hospital, Birmingham, Surgical Casualty Officer—Salary £70 per annum.

Huddersfield Infirmary, Honorary Physician.

New Hospital for Women, 144, Euston Road, N.W., Female Clinical Assistant for In patient Department, fully qualified. Appointment for six months.

Taunton Union, Medical Officer of the Workhouse—Salary £60 per annum, with 10s. extra for midwifery cases.

University of Aberdeen, Six Examiners for Graduation in Medicine.—Grant of £30 per annum each.

### PASS LISTS.

#### Royal College of Surgeons of England.

The following gentleman having passed the necessary examinations, and now attained the legal age (twenty-five years), was admitted a Fellow of the College, namely: Mr. Drew Douglas, M.B.Lond., L.R.C.P.Lond.

The following gentlemen (unless otherwise specified, L.R.C.P.) having passed the necessary examinations, and having conformed to the By-laws and Regulations were admitted Members of the College, viz.:—S. Ali, J. Atcherle, *J. H. E. Austin, J. C. Baker, H. A. Ballance C. W. R. Binham, H. C. Barnes, *H. E. Blake, E. W. Bliss, W. E. Bond, R. A. Bowie, R. W. Brimacombe, H. C. Brown, S. A. Bull, F. V. Bunch, C. Buttar, T. Carwardine, G. W. Cazalet, B. Charles, *R. W. Councell, L.S.A., G. Cross, M. Cutliffe, G. F. Darker, R. Davie, C. A. Dixon, F. Dove, R. H. W. Dunderdale, E. T. P. Eames, F. W. Edwards, J. S. Fowler, H. St. J. Fraser, H. C. French, R. J. Fyffe, A. Gaskell, A. S. Gedge, H. E. Girdlestone, B. J. Gladstone, T. H. J. C. Goodwin, A. Graydon, R. C. Griffith, S. A. E. Griffith, J. Hague, E. M. Hainworth, T. R. Hamlen-Williams, *H. R. Hancock, L.R.C.P.Ire., F. D. Harris, E. B. Hartnell, J. H. Harvey, *F. H. Hawley, L.S.A., A. D. Heath, E. S. Hemsted, R. Henry, M. L. Hepburn, J. D. Hessey, R. H. Hogg, C. S. Jaffee, R. B. James, J. E. M. Jenkins, J. Jones, S. Kent, St. J. B. Killery, M. H. Knapp, M. C. Langford, J. P. Lightfoot, W. C. Loos, C. V. McCormack, G. Mackeson, *W. A. Michie, M.B., Aberdeen, A. E. Milner, A. R. O. Milton, G. F. Morley, J. L. Morton C. B. T. Musgrave, G. F. M. Nellen, H. W. Newton, J. M. Nicol, A. J. Ortlepp, F. A. Osborn, *W. Parker, A. G. Parrott, S. H. Perry, W. A. Perry, W. L. Pethybridge, R. N. U. Pickering, E. Posnett, W. Potter, W. A. Powell, M. Randall, H. F. Ransome, E. G. Renny, C. H. Rock, J. R. Russell, J. L. Sawers, J. G. Scheurer, F. C. Scotson, E. W. Selby, W. Selby, A. E. Shaw, J. H. Sims, E. Smith, N. I. Smith, W. R. Smith, *T. A. B. Soden, L.S.A., *G. Soilleux, M.B.Melbourne, C. G. Spencer, J. H. Sproat, R. E. P. Squibbs, H. E. Staddon, S. L. J. Steggall, J. A. Stewart, A. Strange, W. G. Sutcliffe, M. Swabey, H. M. Sylvester, G. Templeton, R. S. Thomas, H. H. Tipping, N. P. F. Toller, R. C. Tweedy, W. J. N. Vincent, M. H. Vinrace, F. Wall, R. A. Walter, A. R. Walters, F. W. Walters, W. Watkins, F. W. Wesley, W. G. West, E. W. Wheatcroft, J. A. H. White, G. C. W. Williams, H. J. E. H. Williams, H. W. S. Williams, E. H. Willock, J. W. Willson, F. N. Windsor, L. A. Winter, F. E. Withers, S. F. Wright, W. C. H. Wroughton, A. T. Wysard, *J. C. Young, M.D.New York.

* Candidates who have not presented themselves under the Regulations of the Examining Board in England.

